# Association of inflammation and nutrition-based indicators and diabetic foot ulcers: a cross-sectional study and a retrospective study

**DOI:** 10.3389/fendo.2025.1654831

**Published:** 2025-09-16

**Authors:** Hua Chen, Yu Zhou, Jiezhi Dai

**Affiliations:** ^1^ Department of Orthopedic Surgery, Shanghai Sixth People’s Hospital, JiaoTong University, Shanghai, China; ^2^ Department of Orthopedic Surgery, Civil Aviation Hospital of Shanghai, Shanghai, China

**Keywords:** inflammation, nutrition, biomarker, DFU, NHANES, retrospective study

## Abstract

**Background:**

Inflammation and nutrition status have emerged as important factors in impaired wound healing in diabetes. However, the association between inflammation and nutrition-based indicators and diabetic foot ulcer (DFU) has not been reported.

**Methods:**

This study used a cross-sectional study based on the National Health and Nutrition Examination Survey (NHANES) database and a clinical retrospective study to investigate the association between the inflammation and nutrition-based indicators and DFU. We analyzed data from 31,126 individuals in the NHANES data between 1999 and 2004. Inflammation and nutrition-based indicators included neutrophil–albumin ratio (NAR), monocyte–albumin ratio (MAR), red cell distribution width–albumin ratio (RAR), the hemoglobin, albumin, lymphocyte, and platelet (HALP) score, and prognostic nutritional index (PNI). Binary logistic regression on single and multiple variables and restricted cubic spline were conducted to assess the association and nonlinear relationship between these biomarkers and the prevalence of DFU. Subgroup analyses were performed to evaluate the stability of the associations. Additionally, a retrospective study was conducted to further assess the associations between NAR, MAR, RAR, HALP, PNI, and the prevalence of DFU using binary logistic regression analysis.

**Results:**

A total of 129 participants with DFUs and 1,515 without DFUs were included in this cross-sectional study. NAR, MAR, RAR, HALP, PNI, and DFU are significantly associated with the prevalence of DFU. After adjusting for all covariates (model 3), the third tertile of NAR (OR = 1.73 [1.09–2.74]), MAR (OR = 1.71 [1.05–2.79]), and RAR (OR = 4.47 [2.57–7.77]) were positively linked with DFU, compared with the first tertile. The third tertile of HALP (OR = 0.50 [0.31–0.80]) and PNI (OR = 0.42 [0.26–0.67]), respectively, were negatively linked with DFU compared with the first tertile. The RCS curves showed a nonlinear relationship between RAR and the prevalence of DFU, with an inflection point at 3.83. In the retrospective study, NAR, MAR, and RAR were positively associated with the prevalence of DFU as follows: NAR: OR = 4.71 (1.99–11.18), MAR: OR = 2.56 (1.23–5.31), and RAR: OR = 6.15 (2.31–16.41). On the other hand, HALP and PNI were negatively linked with the risk of DFU (HALP: OR = 0.93 [0.90–0.97] and PNI: OR = 0.85 [0.78–0.93]).

**Conclusion:**

High NAR, MAR, and RAR were positively associated with the prevalence of DFU, whereas low HALP and PNI were linked with an increased prevalence of DFU. In addition, RAR performed better in terms of predictive ability.

## Introduction

Diabetic foot ulcer (DFU) is the most serious and costly complication of diabetes (1). It plays a very important role in the occurrence of vascular disease, neuropathy, and infection of diabetes. In severe cases, amputation is required, which significantly affects the patients’ quality of life. Therefore, early identification of DFU was of great importance.

DFU is typically associated with a persistent inflammatory response (2). The levels of inflammatory biomarkers in patients with DFU are significantly increased, including white blood cell count (WBC), C-reactive protein (CRP), procalcitonin, erythrocyte sedimentation rate (ESR), etc. Despite inflammatory biomarkers being closely associated with the diagnosis and prognosis of DFU, they still lack specificity and are influenced by multiple factors. Nutritional status has also been associated with the progression of DFU (3). Malnutrition can prolong the inflammatory phase, limit collagen synthesis, and increase the risk of new wound formation (4). Albumin level is a common and widely used biomarker used to assess malnutrition (5). Previous studies have shown the negative association of DFU with serum albumin levels (6). Therefore, it is significant to explore the link between inflammation, nutritional status, and DFU.

Recently, the relationship between inflammation, nutritional status, and DFU has gained great attention from clinicians. Neutrophil–albumin ratio (NAR), which is the ratio of neutrophil count to albumin value, shows potential in assessing the severity of inflammation and predicting the prognosis in infectious diseases (7). Monocyte–albumin ratio (MAR), calculated from the ratio of monocytes to albumin, reflects systemic inflammation and nutritional status. Red cell distribution width–albumin ratio (RAR) is a comprehensive and innovative inflammatory biomarker based on both red cell distribution width (RDW) and albumin (8). The hemoglobin, albumin, lymphocyte, and platelet (HALP) score is a novel, easily calculated index that combines hemoglobin, albumin, lymphocyte, and platelet counts to provide a comprehensive assessment of both inflammation and nutritional status (9). Prognostic nutritional index (PNI) is calculated using albumin levels and peripheral lymphocyte count, and Coşkun et al. found that PNI was associated with an increase in amputation rate in patients with DFU (10). Despite their utility as indicators of inflammation and nutritional status, these inflammatory and nutritional biomarkers have not been extensively studied in patients with DFU.

The National Health and Nutrition Examination Survey (NHANES) is a nationally representative cross-sectional study aimed at conducting a comprehensive assessment of the health status and nutritional levels of the US population. In this study, we performed a cross-sectional study based on NHANES database and a clinical retrospective study to evaluate the clinical and predictive value of the inflammation and nutrition-based indicators in patients with DFU. Our aim is to clarify the possible function of NAR, MAR, RAR, HALP, and PNI as prognostic biomarkers for DFU by evaluating their levels and examining their clinical outcomes.

## Methods

### Data source

Data of this cross-sectional study were derived from the NHANES database. We included data from three NHANES cycles (1999–2000, 2001–2002, and 2003–2004). The NCHS Ethics Review Board approved the NHANES protocol, and all participants provided informed consent. This study strictly adhered to the ethical standards outlined in the 1964 Declaration of Helsinki and its subsequent revisions. The data were extracted for secondary analysis, obviating the need for additional ethical approval.

The clinical retrospective study involving human participants was reviewed and approved by the Ethics Review Board of Shanghai Sixth People’s Hospital. The patients/participants provided their written informed consent to participate in this study. Written informed consent was obtained from the individual(s) for the publication of any potentially identifiable images or data included in this article.

### Study population

**Figure 1:**
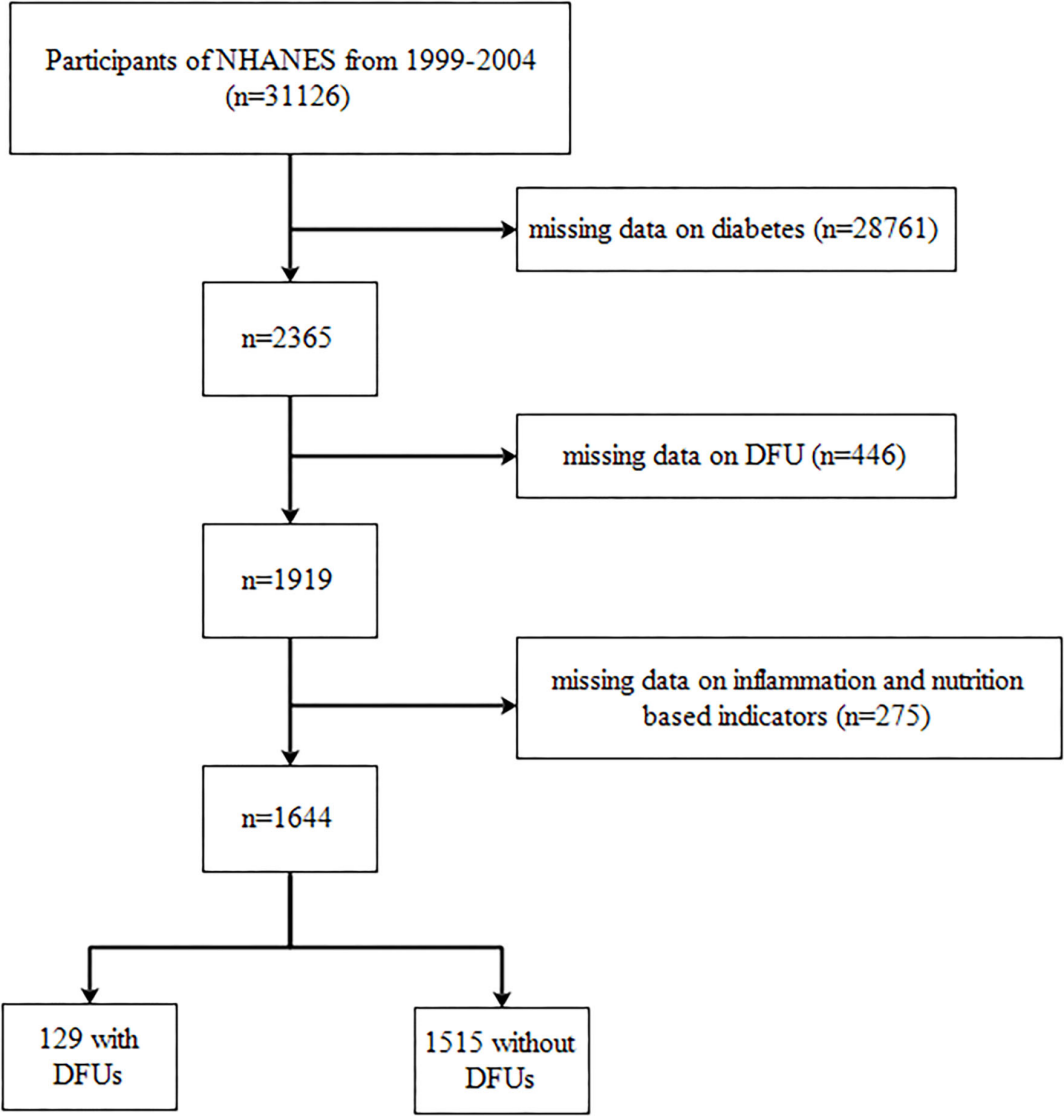
Flowchart of the participants’ selection.

In this cohort study, a total of 31,126 individuals from the NHANES database were initially included. The exclusion criteria included participants with missing data on diabetes (*n* = 28,761), those with missing data on DFU (*n* = 446), and those with missing data on inflammation and nutrition-based indicators (*n* = 275). Consequently, our final analysis comprised 1,644 individuals, including 129 with DFUs and 1,515 without DFUs. We presented the selection process in **Figure 1**.

### Variable determination

The baseline characteristics included age, gender (male or female), race/ethnicity, marital status, education level, body mass index (BMI), smoking status, blood pressure, HbAlc, fasting blood glucose (FBG), CRP, WBC, lymphocyte, monocyte, neutrophil, platelet, hemoglobin (Hb), total cholesterol, HDL-cholesterol, albumin, and red cell distribution width (RDW). Diabetes was defined if they had any one of the following symptoms—(1) FBG ≥126 mg/dL, (2) random blood glucose ≥200 mg/dL, (3) HbA1c ≥6.5%, and (4) doctor told you have diabetes/taking insulin now/taking diabetic pills to lower blood sugar—through the diabetes questionnaire. DFU was defined as an ulcer/sore not healed within 4 weeks through the diabetes questionnaire. Smoking was defined as “smoked at least 100 cigarettes in life” or “do you now smoke cigarettes” through the smoking questionnaire. Hypertension status was defined as if they had any one of the following symptoms—(1) systolic blood pressure average ≥140 mmHg, (2) diastolic blood pressure average ≥90 mmHg, and (3) ever told you had high blood pressure/taking prescription for hypertension—through the blood pressure questionnaire. NAR was calculated as neutrophil count/serum albumin. MAR was calculated as monocyte count/serum albumin. RAR was calculated as RDW percentage/serum albumin (g/dL). HALP was calculated as Hb (g/L) × serum albumin (g/L) × lymphocyte count/platelet count. PNI was calculated as serum albumin (g/L) + 5 × lymphocyte count.

### Statistical analysis

Data processing and analysis were performed using SPSS 18.0, R version 4.3.3, along with Zstats 1.0 (www.zstats.net). Categorical variables were expressed as frequencies and percentages, and continuous variables were expressed as means and standard deviations. Continuous variables were examined with Student’s *t*-test, while categorical variables were tested through the chi-square test. Binary logistic regression on single and multiple variables was conducted to examine the relationship between the inflammation and nutrition-based indicators and DFU. We presented three models for multivariate logistic regression: (1) model 1: unadjusted for any other variables, (2) model 2: adjusted for age, gender, and race/ethnicity, and (3) model 3: adjusted for age, gender, race/ethnicity, marital status, education level, smoking, and hypertension. We used restricted cubic spline curve (RCS) and threshold effects analyses to investigate whether there was a nonlinear link in the abovementioned relationships. Subgroup analyses were performed to evaluate the stability of the associations based on age (<65, ≥65), gender, race/ethnicity, marital status, education level, smoking, and hypertension. *P*-values less than 0.05 were considered statistically significant. The percentage of missing values is less than 5%. To address this issue, missing data for categorical variables were imputed with the highest frequency.

### Clinical study

A retrospective study comparing patients with DFU and newly admitted type 2 diabetes patients without DFU was conducted in Shanghai Sixth People’s Hospital from February 2024 to December 2024. This study was approved by our institutional review board. We included patients with type 2 diabetes according to the diagnostic criteria recommended by the American Diabetes Association in 2010 (11). We excluded type 1 diabetes, rheumatic disease, cardiovascular disease, renal failure, and malignancy. Patients with chronic wounds due to vasculitis, pyoderma gangrenosum, pressure ulcers, or wound infections not related to DM were excluded.

Clinical characteristics included the patients’ age, gender, BMI, HbAlc, CRP, WBC, lymphocyte, monocyte, neutrophil, platelet, Hb, total cholesterol, HDL-cholesterol, albumin, and RDW. Categorical variables were expressed as frequencies and percentages, and continuous variables were expressed as means and standard deviations. Continuous variables were examined with Student’s *t*-test, while categorical variables were tested through the chi-square test. A logistic regression analysis was performed to identify the relationship between NAR, MAR, RAR, HALP, PNI, and the prevalence of DFU. Results with *P*-values less than 0.05 were considered statistically significant.

## Results

### Participants’ characteristics

We presented the participant’s baseline characteristics in **Table 1**. A total of 1,644 participants who met the inclusion criteria were included in this study. The mean age was 64.85 years, and 52.25% were men. Among the participants, 129 (7.8%) had DFU. There were no significant differences in age, gender, race/ethnicity, education level, smoking, hypertension, BMI, CRP, lymphocyte, monocyte, platelet, total cholesterol, HDL-cholesterol, and HbAlc. The participants with DFU had higher levels of neutrophil and RDW and lower levels of Hb and albumin. Regarding the inflammation and nutrition-based indicators, participants with DFU had higher NAR, MAR, and RAR and lower HALP and PNI.

**Table 1:** Baseline characteristics of the study population.

Variable	Total (*n* = 1,644)	DFU (*n* = 129)	Non-DFU (*n* = 1,515)	*P*
Age (years)		65.60 ± 12.14	64.79 ± 11.84	0.458
Gender (%)				0.078
Male	859	77 (59.7%)	782 (51.6%)	
Female	785	52 (40.3%)	733 (48.4%)	
Race				0.623
Mexican American	469	40 (31%)	429 (28.3%)	
Other Hispanic	66	6 (4.7%)	60 (4.0%)	
Non-Hispanic White	681	55 (42.6%)	626 (41.3%)	
Non-Hispanic Black	372	26 (20.2%)	346 (22.8%)	
Other race	56	2 (1.6)	54 (3.6%)	
Education level				0.414
Less than high school	794	60 (46.5%)	734 (48.4%)	
High school	343	23 (17.8%)	320 (21.1%)	
Higher than high school	507	46 (35.7%)	461 (30.4%)	
Marital status				0.046
Married/living with partners	1,020	67 (51.9%)	953 (62.9%)	
Widowed/divorced/separated	530	52 (40.3%)	478 (31.6%)	
Never married	94	10 (7.8%)	84 (5.5%)	
Smoking				0.618
Yes	883	72 (55.8%)	811 (53.5%)	
No	761	57 (44.2%)	704 (46.5%)	
hypertension				0.448
Yes	1,189	97 (75.2%)	1,092 (72.1%)	
No	455	32 (24.8%)	423 (27.9%)	
BMI (kg/m^2^)		31.70 ± 7.78	30.63 ± 6.49	0.078
CRP (mg/dL)		0.85 ± 1.22	0.70 ± 1.50	0.285
WBC (10^9^)		7.78 ± 2.41	7.44 ± 2.14	0.097
Lymphocyte (10^9^)		2.03 ± 0.86	2.17 ± 0.93	0.084
Monocyte (10^9^)		0.61 ± 0.21	0.58 ± 0.19	0.139
Neutrophil (10^9^)		4.84 ± 1.96	4.44 ± 1.62	0.007
Hb (g/dL)		13.68 ± 1.63	14.15 ± 1.58	0.001
Platelet (10^9^)		259.34 ± 75.26	257.50 ± 73.04	0.784
Total cholesterol (mg/dL)		196.57 ± 45.59	203.51 ± 42.51	0.077
HDL-cholesterol (mg/dL)		47.43 ± 16.73	48.17 ± 14.36	0.578
Albumin (g/dL)		4.03 ± 0.35	4.18 ± 0.35	<0.001
RDW (%)		13.44 ± 1.31	13.07 ± 1.31	0.002
HbA1c (%)		7.23 ± 2.31	7.08 ± 1.83	0.437
NAR		1.20 ± 0.49	1.07 ± .042	0.001
MAR		0.15 ± 0.05	0.14 ± 0.05	0.016
RAR		3.37 ± 0.55	3.16 ± 0.50	<0.001
HALP		46.03 ± 23.31	53.32 ± 30.71	0.008
PNI		50.46 ± 6.03	52.66 ± 5.84	<0.001

HBP, high blood pressure; RDW, red cell distribution width; NAR, neutrophil-albumin ratio; MAR, monocyte-albumin ratio; RAR, red cell distribution width-albumin ratio; HALP, hemoglobin, albumin, lymphocyte, and platelet; PNI, prognostic nutritional index.

### Association between the inflammation and nutrition-based indicators and DFU

We presented the relationship between the inflammation and nutrition-based indicators and DFU in **Table 2**. When analyzed in continuous form, a significant correlation between NAR, MAR, RAR, HALP, PNI, and DFU was observed in the unadjusted model l, adjusted model 2, and adjusted model 3.

**Table 2:** Association of NAR, MAR, RAR, HALP, and PNI with the prevalence of DFU.

Variable	Model 1	*P*	Model 2	*P*	Model 3	*P*
	OR (95% CI)		OR (95% CI)		OR (95% CI)	
NAR	1.815 (1.277, 2.581)	0.001	1.832 (1.284, 2.615)	0.001	1.819 (1.271, 2.604)	0.001
NAR tertiles						
Tertiles 1	1.00 (Reference)		1.00 (Reference)		1.00 (Reference)	
Tertiles 2	1.17 (0.73,1.89)	0.518	1.16 (0.72, 1.88)	0.543	1.17 (0.72, 1.90)	0.532
Tertiles 3	1.76 (1.12,2.75)	0.013	1.80 (1.14, 2.83)	0.012	1.73 (1.09, 2.74)	0.020
*P*-trend		0.009		0.008		0.014
MAR	1.449 (1.067, 1.969)	0.018	1.461 (1.072, 1.991)	0.016	1.434 (1.051, 1.957)	0.023
MAR tertiles						
Tertiles 1	1.00 (Reference)		1.00 (Reference)		1.00 (Reference)	
Tertiles 2	1.72 (1.07, 2.78)	0.025	1.73 (1.07, 2.79)	0.026	1.76 (1.09, 2.86)	0.022
Tertiles 3	1.76 (1.09, 2.84)	0.021	1.75 (1.08, 2.84)	0.024	1.71 (1.05, 2.79)	0.033
*P*-trend		0.030		0.034		0.048
RAR	1.828 (1.393, 2.398)	<0.001	1.979 (1.497, 2.616)	<0.001	1.939 (1.461, 2.572)	<0.001
RAR tertiles						
Tertiles 1	1.00 (Reference)		1.00 (Reference)		1.00 (Reference)	
Tertiles 2	2.38 (1.37, 4.14)	0.002	2.62 (1.50, 4.57)	<.001	2.54 (1.45, 4.45)	0.001
Tertiles 3	3.78 (2.23, 6.38)	<.001	4.71 (2.72, 8.16)	<.001	4.47 (2.57, 7.77)	<.001
*P*-trend		<0.001		<0.001		<0.001
HALP	0.987 (0.978, 0.996)	0.004	0.985 (0.976, 0.994)	0.002	0.986 (0.977, 0.995)	0.003
HALP tertiles						
Tertiles 1	1.00 (Reference)		1.00 (Reference)		1.00 (Reference)	
Tertiles 2	0.66 (0.43, 1.00)	0.053	0.64 (0.42, 0.98)	0.040	0.66 (0.43, 1.02)	0.063
Tertiles 3	0.53 (0.34, 0.84)	0.006	0.49 (0.31, 0.78)	0.002	0.50 (0.31, 0.80)	0.003
*P*-trend		0.007		0.003		0.004
PNI	0.932 (0.901, 0.963)	<0.001	0.930 (0.899, 0.962)	<0.001	0.935 (0.903, 0.967)	<0.001
PNI tertiles						
Tertiles 1	1.00 (Reference)		1.00 (Reference)		1.00 (Reference)	
Tertiles 2	0.56 (0.36, 0.85)	0.006	0.54 (0.35, 0.83)	0.005	0.54 (0.35, 0.84)	0.005
Tertiles 3	0.42 (0.26, 0.66)	<0.001	0.41 (0.26, 0.65)	<0.001	0.42 (0.26, 0.67)	<0.001
*P*-trend		<0.001		<0.001		<0.001

Model 1, unadjusted model; Model 2, adjusted for sex, age, and race; Model 3, adjusted for sex, age, race, educational level, marital status, smoking, and hypertension.

We divided NAR, MAR, RAR, HALP, and PNI into tertiles. In model 1, the risk of DFU among those in the third tertile compared with those in the first tertile was increased by 76% (OR = 1.76 [1.12–2.75]) for NAR, 76% (OR = 1.76 [1.09–2.84]) for MAR, and 278% (OR = 3.78 [2.23–6.38]) for RAR. The risk of DFU among those in the third tertile compared with those in the first tertile was decreased by 47% (OR = 0.53 [0.34–0.84]) for HALP and 58% (OR = 0.42 [0.26–0.66]) for PNI. After adjusting for all covariates (model 3), the third tertile of NAR (OR = 1.73 [1.09–2.74]), MAR (OR = 1.71 [1.05–2.79]), and RAR (OR = 4.47 [2.57–7.77]) were positively linked with DFU compared with the first tertile. The third tertile of HALP (OR = 0.50 [0.31–0.80]) and PNI (OR = 0.42 [0.26–0.67]), respectively, were negatively linked with DFU compared with the first tertile.

**Figure 2:**
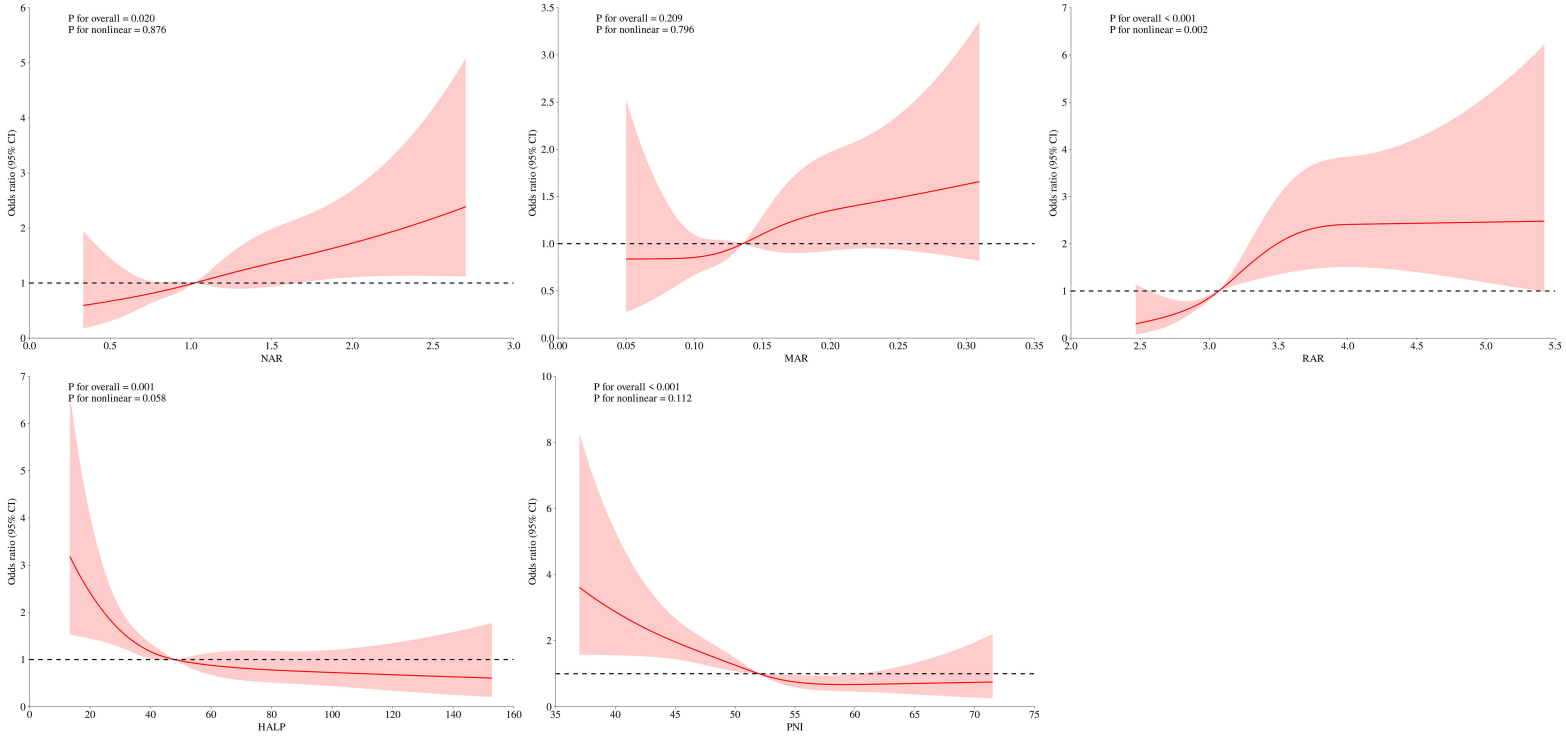
RCS analysis of NAR, MAR, RAR, HALP, and PNI with the prevalence of DFU.

The RCS analyses showed that the relationship between NAR, MAR, HALP, PNI, and DFU was linear (*P* for nonlinearity >0.05), whereas the relationship between RAR and DFU was nonlinear (*P* for nonlinearity <0.05) (**Figure 2**). The threshold effects analyses indicated that the inflection point of RAR was 3.83 (**Table 3**). When RAR was less than 3.83, the risk of DFU escalated with an increasing ratio. Conversely, when the ratio exceeded 3.83, the association between RAR and DFU was not statistically significant.

**Table 3:** Threshold effect analysis of RAR on DFU.

Outcome	Effect	*P*
Model 1: Fitting model by standard linear regression	1.96 (1.47–2.63)	<0.001
Model 2: Fitting model by two-piecewise linear regression		
Inflection point	3.83	
<3.83	5.55 (2.87–10.75)	<0.001
≥3.83	0.89 (0.36–2.20)	0.803
*P* for likelihood test		<0.001

RAR, red cell distribution width–albumin ratio.

### Subgroup analysis

**Figure 3:**
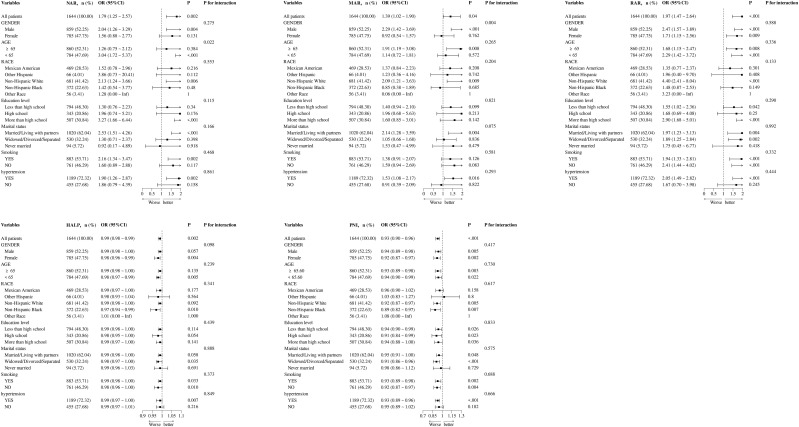
Subgroup analysis for the association between NAR, MAR, RAR, HALP, and PNI with the prevalence of DFU.

Subgroup analyses were performed to determine the potential effect modifications on the relationship between NAR, MAR, RAR, HALP, and PNI and the prevalence of DFU. The relationships between RAR, HALP, PNI, and DFU were not influenced by age, gender, race, education level, marital status, smoking, and hypertension. However, a significant interaction effect was observed in the age subgroup for NAR (*P*-value for interaction = 0.022) and in the gender subgroup for MAR (*P*-value for interaction = 0.004) (**Figure 3**).

### Clinical study

In this retrospective study, 36 patients with DFU and 49 diabetic patients without DFU were included. There was no significant difference in age, BMI, lymphocyte, monocyte, RDW, and total cholesterol. Compared with the non-DFU group, patients with DFU had higher levels of CRP, WBC, neutrophil, Hb, platelet, and RDW and lower levels of HDL-cholesterol and albumin. Regarding the inflammation and nutrition-based indicators, patients with DFU had higher NAR, MAR, and RAR and lower HALP and PNI (**Table 4**).

**Table 4:** Clinical characteristics of the retrospective study.

Variable	DFU (*n* = 36)	Non-DFU (*n* = 48)	*P*
Age (years)	66.86 ± 10.02	70.85 ± 8.71	0.055
Gender (%)			0.006
Male	28 (77.8%)	23 (47.9%)	
Female	8 (22.2%)	25 (52.1%)	
BMI (kg/m^2^)	24.36 ± 3.39	25.18 ± 3.46	0.280
CRP (mg/dL)	40.91 ± 64.02	2.73 ± 5.77	<0.001
WBC (10^9^)	8.82 ± 4.46	6.36 ± 2.66	0.002
Lymphocyte (10^9^)	1.50 ± 0.61	1.63 ± 0.60	0.353
Monocyte (10^9^)	0.56 ± 0.26	0.50 ± 0.17	0.211
Neutrophil (10^9^)	6.59 ± 4.49	4.02 ± 2.20	0.001
Hb (g/dL)	11.47 ± 2.18	13.21 ± 1.64	<0.001
Platelet (10^9^)	265.50 ± 69.54	198.13 ± 62.68	<0.001
Total cholesterol (mg/dL)	152.83 ± 56.50	148.55 ± 50.17	0.715
HDL-cholesterol (mg/dL)	35.51 ± 10.22	43.90 ± 10.88	0.001
Albumin (g/dL)	3.36 ± 0.52	3.91 ± 0.39	<0.001
RDW (%)	13.69 ± 1.91	13.22 ± 1.31	0.188
HbA1c (%)	8.67 ± 2.13	7.15 ± 1.47	0.001
NAR	2.08 ± 1.73	1.03 ± 0.56	<0.001
MAR	0.18 ± 0.10	0.13 ± 0.04	0.005
RAR	4.18 ± 0.91	3.41 ± 0.49	<0.001
HALP	24.71 ± 18.32	45.22 ± 19.31	<0.001
PNI	41.11 ± 6.85	47.28 ± 5.71	<0.001

RDW, red cell distribution width; NAR, neutrophil–albumin ratio; MAR, monocyte–albumin ratio; RAR, red cell distribution width–albumin ratio; HALP, hemoglobin, albumin, lymphocyte, and platelet; PNI, prognostic nutritional index.

The associations of NAR, MAR, RAR, HALP, and PNI with the dependent variable DFU were examined in the logistic regression analyses (**Table 5**). The results showed that NAR, MAR, and RAR were positively associated with the prevalence of DFU as follows: NAR: OR = 4.71 (1.99–11.18), MAR: OR = 2.56 (1.23–5.31), and RAR: OR = 6.15 (2.31–16.41). On the other hand, HALP and PNI were negatively linked with the risk of DFU (HALP: OR = 0.93 [0.90–0.97] and PNI: OR = 0.85 [0.78–0.93]).

**Table 5:** Logistic regression predicting the likelihood of DFU on NAR, MAR, RAR, HALP, and PNI.

Variables	*β*	S.e.	*Z*	*P*	OR (95% CI)
NAR	1.55	0.44	3.52	<0.001	4.71 (1.99–11.18)
MAR	0.94	0.37	2.51	0.012	2.56 (1.23–5.31)
RAR	1.82	0.50	3.63	<0.001	6.15 (2.31–16.41)
HALP	-0.07	0.02	-3.86	<0.001	0.93 (0.90–0.97)
PNI	-0.17	0.04	-3.68	<0.001	0.85 (0.78–0.93)

NAR, neutrophil–albumin ratio; MAR, monocyte–albumin ratio; RAR, red cell distribution width–albumin ratio; HALP, hemoglobin, albumin, lymphocyte, and platelet; PNI, prognostic nutritional index.

## Discussion

In our study, we discussed the relationship between various inflammation and nutrition-based indicators and DFU using both NHANES data and a retrospective analysis. The cross-sectional study suggested that certain biomarkers such as NAR, MAR, RAR, HALP, and PNI were associated with the prevalence of DUF. Higher levels of NAR, MAR, and RAR were associated with an increased prevalence of DFU, while lower levels of HALP and PNI were linked with an increased prevalence of DFU. The results of RCS showed linear associations between NAR, MAR, HALP, PNI, and DFU, whereas the relationship between RAR and DFU was nonlinear. We further performed a retrospective study based on the clinical cases to clarify the relationship between NAR, MAR, RAR, HALP, and PNI with the prevalence of DFU. Similar to the results of the cross-sectional study, we found that NAR, MAR, RAR, HALP, and PNI were significantly associated with the prevalence of DFU. Overall, our findings underscored the importance of monitoring and managing inflammation and nutritional status in participants with DFU.

Inflammation and nutrition status have emerged as important factors in impaired wound healing in diabetes. Recent studies have emphasized the importance of chronic inflammation, which is maintaining a pro-inflammatory environment dominated by cytokines, such as TNF-α, IL-1β, and IL-6, and impairing angiogenesis and delaying wound repair (12). Malnutrition can prolong the inflammatory phase, limit collagen synthesis, and increase the risk of new wound formation (4). In the previous cross-sectional studies from the NHANES database, the inflammation and nutrition biomarkers such as SIRI and anemia have been proven to have a positive correlation with the prevalence of DFU (13, 14). However, it is insufficient to evaluate the occurrence of DFU only based on inflammation or malnutrition. A study has proved that inflammation negatively impacts nutritional status through multiple pathways, such as TNF-α and CRP (15). On the other hand, nutritional status like low albumin and vitamin D deficiency can trigger exaggerated immune responses, prolonging inflammation (16). There is an urgent need for a new and comprehensive indicator to effectively evaluate the correlation between inflammation, nutritional status, and DFU.

NAR is the ratio of neutrophil to albumin, and MAR is the ratio of monocytes to albumin. Both are novel inflammatory biomarkers, mainly reflecting the balance between systemic inflammation and nutritional status. The chronic hyperglycemic state of diabetes has been implicated in impaired neutrophil functions, which prolong the inflammatory phase and disrupt the delicate balance required for effective wound healing (17). In DFUs, an elevated neutrophil count is generally observed. High neutrophil production causes increased NETosis and leads to subsequent delays in wound healing (18). Monocytes are the major source of pro-inflammatory cytokines (IL-1β, IL-6, and TNF-α) as well as anti-inflammatory cytokines (IL-4 and IL-10), which play a key role in the development and maintenance of the inflammatory response (19). The correct timing, intensity, and balance changes in the expression of pro-inflammatory and anti-inflammatory cytokines in monocytes result in the pathologic regulation of the inflammatory response. DFU is characterized by low-grade systemic inflammation, which may cause altered recruitment and an increased presence of myeloid cells (monocytes and neutrophils) at the wound site (20). In addition, protein deficiency has been demonstrated to contribute to poor healing rates with reduced collagen formation and wound dehiscence (21). Evaluating both serum inflammation levels and nutrition can provide valuable insights into early steps to develop a treatment strategy and predict the prognosis of DFU patients. In our cross-sectional study, compared with the first tertile, DFU prevalence increased by 73% and 71% in the third tertile of NAR and MAR, respectively. In the retrospective study, NAR (OR = 4.71 [1.99–11.18]) and MAR (OR = 2.56 [1.23–5.31]) were positively linked with the prevalence of DFU. Both of the two biomarkers show a high correlation with the prevalence of DFU.

RAR is defined as the ratio of RDW to albumin. Elevated RDW levels suggest an imbalance in red blood cells stemming from impaired erythropoiesis and abnormal red blood cell survival (8). Previous studies have shown that RAR is a new combined parameter that can predict mortality in patients with burn surgery (22), diabetic retinopathy (23), and diabetic ketoacidosis (24). Chronic wounds are normally associated with a persistent inflammatory response, which contributes to an increased RDW through myelosuppression. This imbalance can promote erythrocyte apoptosis and erythropoietin resistance and reduce erythropoietin production and the bioavailability of iron (25). In addition, hyperglycemia induces oxidative stress, leading to red blood cell damage and an increase in the heterogeneity of red blood cell volume distribution. In this regard, high RDW and low albumin may be a marker of poor general health and healing abilities of patients with diabetic foot ulcers (26). In the cross-sectional study, compared with the first tertile, the prevalence of DFU increased by 374% in the third tertile of RAR. In the retrospective study, the odds ratios of RAR for the risk of DFU was 6.15 (95% CI: 2.31–16.41). These results indicated a high correlation between RAR and the prevalence of DFU. In addition, a nonlinear correlation between RAR and the prevalence of DFU was observed by RCS analysis. The RCS curves showed an L-shape relationship between RAR and the prevalence of DFU, with an inflection point at 3.83. The RAR was positively correlated with the prevalence of DFU, when RAR was less than 3.83. In the clinical setting, these findings underscore the value of RAR as an indicator for risk of DFU.

HALP, an immune nutritional indicator, was initially introduced by Chen et al. as a scoring system to predict the prognosis of gastric cancer (27). It has been used to assess the relationship with the prevalence of non-neoplastic disease. Ding et al. found that a lower HALP (≤42.9) score was an independent risk factor for diabetic retinopathy (28). Zhao et al. reported that the HALP score was negatively correlated with both all-cause and CVD mortality risk in patients with diabetes or pre-diabetes (29). Each component of the HALP score—hemoglobin, albumin, lymphocytes, and platelets—plays a key role in the development and prognosis of DFU. Low levels of hemoglobin are thought to aggravate lower limb ischemia owing to reduced blood oxygen (30). It has also been reported to cause thrombosis by inducing a hyperkinetic circulatory state and upregulating the endothelial adhesion molecule genes (31). Albumin is essential for collagen formation, angiogenesis, and cellular regeneration, all of which are crucial for wound healing (32). Albumin has antioxidant and anti-inflammatory properties, and low levels can trigger a chronic inflammatory response (33). Lymphocytes are important regulators of inflammation and wound healing progression. Lymphocytes obtained from DFU patients showed an accumulation of ROS, membrane damage, increased protein carbonyls, and altered SOD and catalase activity (34). An activated platelet not only releases inflammatory mediators but also promotes thrombosis by adhering to damaged vascular endothelium, resulting in atherosclerosis and local ischemia (35). PNI provided the nutritional and inflammation status of patients based on albumin levels and lymphocytes. Sun et al. found that patients with DFU-induced sepsis had a significantly lower PNI than those without sepsis (36). Yılmaz et al. reported that PNI had a significant predictive value for 30-day mortality after below-knee amputation in DFU patients (37). In our cross-sectional study, the prevalence of DFU decreased by 47% and 58% in the third tertile of HALP and PNI, respectively. In the retrospective study, HALP (HALP: OR = 0.93 [0.90–0.97]) and PNI (OR = 0.85 [0.78–0.93]) were negatively linked with the risk of DFU.

To our knowledge, this is the first study to demonstrate a relationship between NAR, MAR, RAR, HALP, and PNI and the prevalence of DFU. As comprehensive biomarkers, NAR, MAR, RAR, HALP, and PNI reflect both systemic inflammatory responses and nutritional status, two factors that are critically involved in the progression of DFU. We demonstrated that these inflammation and nutrition-based indicators were significantly associated with the prevalence of DFU; especially RAR performed better predictive ability. All of these biomarkers rely on direct laboratory tests and have the advantages of being easy to use and inexpensive, which make them potentially applicable in various clinical settings.

This study combined a cross-sectional study and an observational, retrospective study, which enrolled DFU cases from individuals (NHANES) and hospitalized patients, offering a comprehensive view of the potential relationships between the inflammation and nutrition-based indicators and DFU. This study has several advantages: (1) the cross-sectional study utilizes the largest population-level data based on the NHANES database, (2) multiple statistical methods, including multivariate adjustment, RCS, and threshold effects analyses and subgroup analyses were employed to increase the credibility and authenticity of our conclusions, and (3) an observational, retrospective study was conducted to strengthen the validity of our findings.

Despite the strengths of this study, several limitations should be considered. First, the cross-sectional design of NHANES limits our ability to capture longitudinal changes or respond to intervention measures. Second, some data in the cross-sectional study was obtained through family interviews and surveys, which raise the possibility of self-report bias or recall bias. Third, the cross-sectional study used a representative sample of the U.S. population, and our retrospective study was primarily based on Chinese population. It is very important to conduct further researches involving a broader, more diverse population. In addition, the sample size included in this retrospective study was too small, and further large-scale studies are necessary. Finally, despite adjusting for known factors such as age, gender, race, education level, marital status, smoking, and hypertension, potential confounding factors may still exist. More well-designed and large-sample studies are needed in the future.

## Conclusion

This study used a cross-sectional study based on NHANES database and a clinical retrospective study to investigate the association between the inflammation and nutrition-based indicators and DFU. It concluded that inflammation and nutrition-based indicators are significantly associated with the prevalence of DFU. High NAR, MAR, and RAR, respectively, were positively associated with the prevalence of DFU, whereas low HALP and PNI were linked with an increased prevalence of DFU. In addition, RAR performed better predictive ability. Continuous and dynamic monitoring of inflammation and nutritional status may contribute to the early diagnosis and treatment of DFU.

## Data Availability

The original contributions presented in the study are included in the article/Supplementary Material. Further inquiries can be directed to the corresponding authors.
